# Protective Role of Surfactant Protein D in Ocular *Staphylococcus aureus* Infection

**DOI:** 10.1371/journal.pone.0138597

**Published:** 2015-09-23

**Authors:** Zhiyong Zhang, Osama Abdel-Razek, Samuel Hawgood, Guirong Wang

**Affiliations:** 1 Department of Surgery, The State University of New York, Upstate Medical University, Syracuse, New York, United States of America; 2 Departments of Ophthalmology, Zhejiang Medical College Affiliated Zhejiang Hospital, Hangzhou, Zhejiang, P. R. China; 3 Department of Pediatrics and the Cardiovascular Research Institute, University of California, San Francisco, California, United States of America; University of Pittsburgh, UNITED STATES

## Abstract

*Staphylococcus aureus* is one of the most common pathogens causing keratitis. Surfactant protein D (SP-D) plays a critical role in host defense and innate immunity. In order to investigate the role of SP-D in ocular *S*. *aureus* infection, the eyes of wild-type (WT) and SP-D knockout (SP-D KO) C57BL/6 mice were infected with *S*. *aureus* (10^7^ CFU/eye) in the presence and absence of cysteine protease inhibitor(E_64_).Bacterial counts in the ocular surface were examined 3, 6, 12, 24 hrs after infection. Bacterial phagocytosis by neutrophils and bacterial invasion in ocular epithelial cells were evaluated quantitatively. *S*. *aureus*-induced ocular injury was determined with corneal fluorescein staining. The results demonstrated that SP-D is expressed in ocular surface epithelium and the lacrimal gland; WT mice had increased clearance of *S*. *aureus* from the ocular surface (p<0.05) and reduced ocular injury compared with SP-D KO mice. The protective effects of SP-D include increased bacterial phagocytosis by neutrophils (p<0.05) and decreased bacterial invasion into epithelial cells (p<0.05) in WT mice compared to in SP-D KO mice. In the presence of inhibitor (E_64_), WT mice showed enhanced bacterial clearance (p<0.05) and reduced ocular injury compared to absent E_64_ while SP-D KO mice did not. Collectively, we concluded that SP-D protects the ocular surface from *S*. *aureus* infection but cysteine protease impairs SP-D function in this murine model, and that cysteine protease inhibitor may be a potential therapeutic agent in *S*. *aureus* keratitis.

## Introduction

*Staphylococcus aureus* is one of the most important pathogens causing keratitis, a disease that can lead to serious vision loss [1,2]. The mucin layer and intercellular tight junctions of corneal epithelium are the main two barriers that prevent *S*. *aureus from* binding to and penetrating into the cornea. A disruption of these barriers can significantly increase the susceptibility to *S*. *aureus* infection and results in *S*. *aureus* keratitis [3,4]. The innate immune system, other than the barriers mentioned above, is the first-line defense that contributes to maintaining a healthy ocular surface [5–7]. An efficient innate immune system is critical for corneal protection from potential pathogens and other environmental factors, however the innate immunity in the eye is not well understood yet. A recent study demonstrated that surfactant protein D (SP-D), a member of C-type collectin family, could protect corneal epithelial cells against invasion and cytotoxicity by the Gram-negative bacteria *Pseudomonas aeruginosa* [8].

SP-D, first identified in the lung, is one of four surfactant-associated proteins and consists of four functional domains: the amino terminal domain, collagen-like domain, neck region, and carbohydrate recognition domain (CRD) [9]. SP-D is hydrophilic protein and maintains alveolar integrity as well as plays a critical role in host defense, regulation of inflammation, and surfactant homeostasis in the lung [10,11]. In addition to the surfactant-related role, SP-D functions as an important innate immune molecule that can bind to carbohydrates and lipids on the surfaces of various microorganisms and cause bacterial aggregation, and enhance the host’s capacity to clear a variety of pathogens as well as allergens [10–12]. SP-D protein also interacts with several receptors on the surface of phagocytic and inflammatory cells in a calcium-dependent manner, and acts as an opsonin to accelerate microbial clearance [13,14]. In some instances it is directly antimicrobial and influences the immune response by activation of complement as well as regulation of macrophage and lymphocyte activities [12,15,16]. SP-D deficient mice are highly susceptible to viral and bacterial infections caused by *S*. *aureus*, *Pseudomonas aeruginosa*, *Haemophilus infuenzae* and respiratory syncytial virus [17–20]. Although SP-D is predominantly expressed in the lung, it has been found in several extrapulmonary tissues/fluids including tear fluid, salivary gland, ovary, uterus, esophagus and heart [21–23]. Recently, the expression of SP-D was detected in healthy larcrimal glands, nasol arcrimal duct and tear fluid in patients with herpetic keratitis. Corneal ulceration surrounding lesions expressed higher level of SP-D than that in healthy cornea, suggesting that SP-D plays a protective role in ocular surface infection [23].

The major extracellular proteases secreted by *S*. *aureus*, such as Staphopains A, cysteine protease, serine protease and metalloprotease, appear to be essential for successful bacterial infection and survival in host. These proteases are activated in a proteolytic cascade; they then interact with the host tissue components and modulate host defense mechanisms [24–30]. Thus, the objective of this study is to investigate SP-D’s role in the first-line host defense in ocular *S*. *aureus* infection and the influence of cysteine protease, one major *S*. *aureus* protease, in a murine ocular infection model.

## Materials and Methods

### Bacterial culture

A *S*. *aureus* strain (ATCC 25923) was purchased from the American Type Culture Collection (Manassas, VA). Bacteria were grown overnight (16–18 h) on tryptic soy agar plates at 37°C before suspension in a sterile phosphate-buffered saline to a bacterial concentration of 2x10^9^ colony-forming units (CFU)/ml [31]. The bacterial suspension was then used for inoculation immediately. The CFU per milliliter value was determined by counting the number of colonies on solid agar plates. To study the clearance of bacteria in ocular surface, tear fluid was collected by capillary action using a 10-μl volume glass capillary tube (Drummond Scientific Co., Broomall, PA) from the lateral canthus after 5 μl of PBS was added to the ocular surface. Viable bacteria in tear fluids were assessed using quantitative plating [8].

### Mouse model

WT C57BL/6 mice (The Jackson Laboratory, Bar Harbor, ME), and SP-D knockout (SP-D KO) mice on the C57BL/6 background were used at 8–12 weeks of age. Original SP-D KO mice were generated as described previously [32]; Mice were bred to homozygosity and backcrossed more than 10 generations onto the C57BL/6 background as described previously [33]. Animals were maintained under pathogen-free housing conditions and fed rodent chow and autoclaved water *ad libitum*. SP-D KO mice were propagated and raised in the animal core facility of SUNY Upstate Medical University [32]. All experiments involved 8 ~ 12 animals per group and were repeated at least twice.

The murine ocular infection model was performed as described previously [8]. Briefly, the ocular surface of anesthetized mice (intraperitoneal injection with ketamine/xylazine (90 mg/kg ketamine, 10 mg/kg xylazine) was inoculated with a 5 μl suspension containing 10^7^ CFU bacteria per eye. Each hour after inoculation, the animals were monitored. At 3, 6, 12 and 24 h post-inoculation, the number of viable bacteria within tear fluid was determined by counting the number of colonies on solid agar plates. In some experiments, 5 μl of 10^7^ CFU bacteria and 5 μl of 10^7^ CFU bacteria with 10 nM of selective synthetic cysteine protease inhibitor (E_64,_transepoxysuccinyl-L-leucylamido-(4-guanido)-butane, Sigma-Aldrich, St. Louis, MO) were applied to the right and left eye of each animal, respectively [34].Animals were sacrificed 24 h after inoculation, and lacrimal glands, upper eye lids with conjunctiva, and eyeballs were removed and processed for immunohistochemistry and SP-D gene knockout mice confirmation. At sacrifice, mice were anesthetized with ketamine/xylazine intraperitoneal injection (90 mg/kg ketamine, 10 mg/kg xylazine). Following adequate anesthesia (as assessed by complete suppression of pedal and ocular reflexes) the abdomen of the animals was opened via a midline incision and exsanguinated by transecting the vena cava and aorta. All procedures involving animals were performed in strict accordance with the ARVO Statement for the Use of Animals in Ophthalmic and Vision Research and the recommendations in the Guide for the Care and Use of Laboratory Animals of the National Institutes of Health. The protocol was approved by the Institutional Animal Care and Use Committee at The State University of New York, Upstate Medical University (IACUC #270) and meets ARRIVE guidelines on the use of laboratory animals.

To determine whether *S*. *aureus* was directly killed by E_64_, 10 nM of E_64_ was prepared by dissolving E_64_ in sterile PBS. 10^7^ CFUs of the bacteria was incubated in PBS with 10 nM of E_64_ at room temperature for 3 and 6 h, and total CFUs of Viable bacteria were analyzed using quantitative plating.

### SP-D null mice confirmation

SP-D gene deficiency in SP-D KO mice was confirmed by PCR analysis with DNA from eye tissues using mouse SP-D primers: forward (GW122), 5′-AGTTGGAGGCTTGCAGTGTGATTG-3′; reverse (GW123), 5′-GGCCCATGGGACCTACCGAGTG-3′. PCR conditions were 30 sec at 95°C, 30 sec at 58°C, and 30 sec at 68°C for 35 cycles after an initial denaturing step of 5 min at 95°C, and then 7 min at 68°C for extension. The PCR products were examined by 1% agarose gel electrophoresis. WT mice contain 0.5 Kb PCR products but SP-D KO mice do not.

### Corneal fluorescein staining

To evaluate the ocular injury caused by *S*. *aureus* on the ocular surface, corneal fluorescein staining was performed with slit-lamp biomicroscopy under cobalt blue light 3 min. after instillation of 1 μl of 1% sodium fluorescein (Sigma–Aldrich, St. Louis, MO) to the inferior-lateral conjunctive sac of the mice. Mouse corneas were photographed before and after staining [35–38].Staining for each of the five corneal zones (i.e., superior, inferior, temporal, nasal, and central) was scored as previously reported [39]. Briefly, the fluorescein-stained corneal area was scored for each zone from 0 (absent) to 3 (diffuse loss of epithelium) [39].

### Bacterial invasion index of epithelial cells

To determine the bacterial burden in an individual epithelial cell, we utilized an invasion index to represent the proportion of infected cells and how heavily each cell was colonized [40,41].To calculate the invasion index in epithelial cells, tear fluid was collected from the ocular surface bacterial inoculation in the absence or presence of 10 nM of E_64_, for 6 h, the cells was sedimented by centrifugation at 4°C at 250xg for 10 min and washed three times with cold PBS followed by sedimentation. The final suspension was resuspended in 200 μl of PBS and the cells were mounted on slides by cytospin centrifugation. The slides were stained using the Hema-3 Stain Kit (Fisher Scientific, Pittsburgh, PA). With light microscopy, one hundred randomly selected epithelia per slide were analyzed at ×1,000 magnification. Bacteria at adherence and internalization steps were counted as invasion bacteria [35]. The invasion index was calculated and expressed as the ratio of invasion bacteria to total bacterium-laden epithelial cells. The percentage of epithelial cells invaded by *S*. *aureus* was also calculated.

### Phagocytic index of neutrophils

To study phagocytosis of *S*. *aureus* by neutrophils we assessed the phagocytic index (PI) by neutrophils. After inoculation with bacteria in the absence or presence of 10 nM of E_64_ for 6 h, mouse tear fluid was collected from ocular surface as described above. After slides were stained, one hundred randomly selected neutrophils per slide were analyzed at ×1,000 magnification. The PI was calculated as the percent of bacteria-positive neutrophils (cells that phagocytized at least one bacterium) multiplied by the average number of bacteria per bacteria-positive neutrophil [41].

### Immunohistochemistry (IHC)

For analysis by immunohistochemistry with SP-D antibody, lacrimal glands, upper eye lids with conjunctiva, and corneas from WT and SP-D KO mice were fixed in 10% formalin for at least 24 h and embedded in paraffin [42]. About 4-μm sections from six mice for each condition were analyzed. Xylene deparaffinised sections were washed in 100% ethanol, rehydrated in graded ethanol, and incubated with 3% hydrogen peroxide in methanol for 20 min to block endogenous peroxidase activity. Antigen retrieval was performed by boiling in 0.01 M citrate buffer (pH 6) for 20 min. Nonspecific binding was inhibited by incubation with 0.5% goat serum in PBS and 0.3% BSA for 2 h at room temperature. Analysis using an ABC kit (Vector Laboratories, Inc., Burlingame CA) was performed by incubating in primary antibody overnight at 4°C (rabbit anti-mouse SP-D, diluted 1:1000) and then secondary antibody (goat anti- rabbit IgG, diluted 1:500 in PBS) for 1 h. For negative control, the same protocol was applied, but the primary antibody was replaced with isotype-matched goat IgG. Sections of lung tissue were used for positive control. The bound antibody was then visualized by peroxidase-labeled streptavidin-biotin and 3,3′-diaminobenzidine (DAB) for at least 5 min. After counterstaining with hemalum, the sections were examined under a light microscope.

To confirm the epithelial cells in tear fluid, IHC analyses were performed with pancytokeratins AE1/AE3 antibody [43,44]. Cells in tear fluid were collected and mounted on slides as described above. The cells were fixed with 4%paraformaldehyde for 10 min at room temperature. To perform IHC, the slides were washed with PBS and sequentially incubated at room temperature in PBS containing goat serum for 10 min, and then monoclonal pancytokeratin AE1/AE3 primary antibody at 1:50 dilution (DakoCytomation, Carpinteria, CA) for 1h [44]. After the reaction with the primary antibodies, the cells were incubated with biotinylated goat anti-rabbit IgG secondary antibodies. The reaction was visualized by peroxidase-labeled streptavidin-biotin and DAB. Finally, the slides counterstained with hemalum. As positive and negative controls, sections of murine skins and lymph nodes were used. Immuno-reactivity was detected microscopically.

### Western blotting analysis

To examine the level of SP-D in murine tear fluid, tear fluid was harvested by washing the ocular surface of anesthetized mice with 5 μl of sterile PBS. The samples were pooled from 5 mice per group. The conditioned medium of bacterial culture was prepared by centrifugation at 2000×g for 5 min of overnight *S*. *aureus* liquid medium culture. SP-D was detected by Western blotting analysis, using a specific SP-D antibody as described previously [23,45,46]. In brief, total protein concentration was determined with a BCA assay (Pierce, Rockford, IL), and equivalent amount of protein was subjected to gel electrophoresis (10% sodium dodecyl sulfate-polyacrylamide gel electrophoresis precast Tris-HCl polyacrylamide gel, SDS-PAGE). The protein in the gel was transferred onto a polyvinylidene difluoride membrane. SP-D was detected using a rabbit antibody (IgG) to rat SP-D at a 1:3000 dilution and then goat anti-rabbit IgG (horseradish peroxidase-conjugated) antibody. The blot was exposed to XAR film following enhanced chemiluminescent detection. Mouse SP-D from WT mouse BAL fluid was used as a positive control in the assay.

### Statistical analysis

All data are expressed as means ±SE or interquartile rang (IQR). Log 10-transformed colony count data were used in statistical analysis. In the experiments of examining the clearance of *S*. *aureus* in the tear fluid of WT and KO mice, data from both eyes of each animal were averaged and used for statistical analysis. Data from one eye of each animal for examining the effects of E_64_ on the clearance of *S*. *aureus* in ocular surface were used as one data point in statistical analysis. Statistical analysis was performed using the SigmaStat (version 3.5., Jandel Scientific, CA). Differences between/among groups were assessed by student’s t test or Mann-Whitney U test, where appropriate. Significant differences were considered at p< 0.05.

## Results

### Analysis of SP-D expression in the ocular tissues

**Fig 1:**
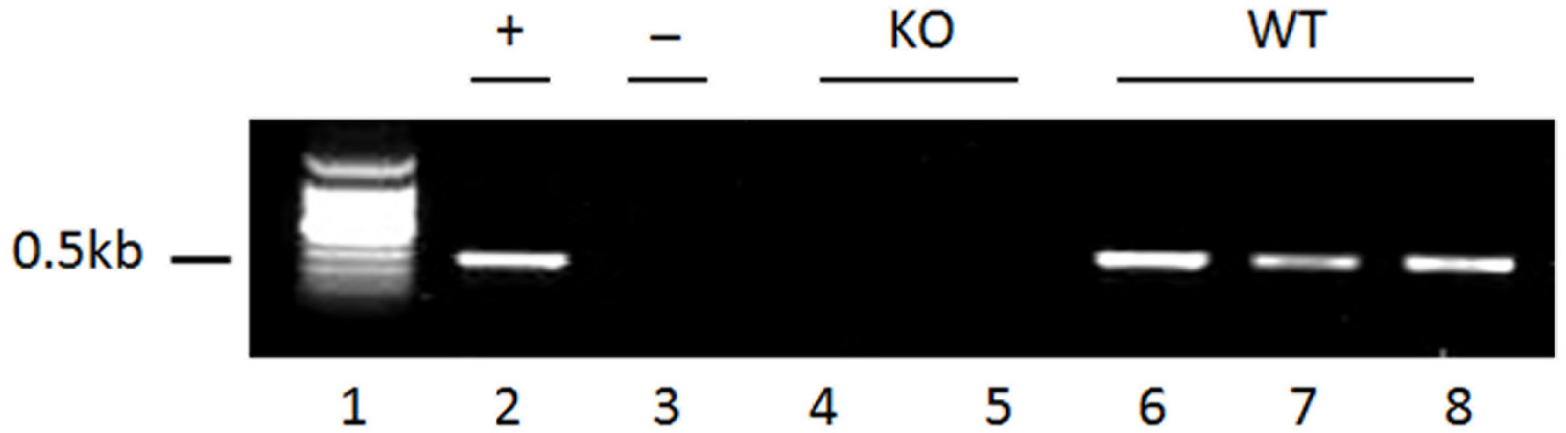
Mouse SP-D-deficient confirmation in eye tissue. SP-D gene deficiency was confirmed by PCR analysis with DNA from the lacrimal gland. Primers (GW122/GW123) were used to detect the exon 2 of mouse SP-D gene. WT mice contain 0.5 Kb PCR products but SP-D KO mice do not have the products. KO, SP-D KO mice (lanes 4, 5); WT, WT mice (lanes 6–8); Lanes 2 and 3 are positive (tail DNA from WT mice) and negative (tail DNA from KO mice) control, respectively; Lane 1 is DNA marker. The figure is from representative results in three independent experiments.

**Fig 2:**
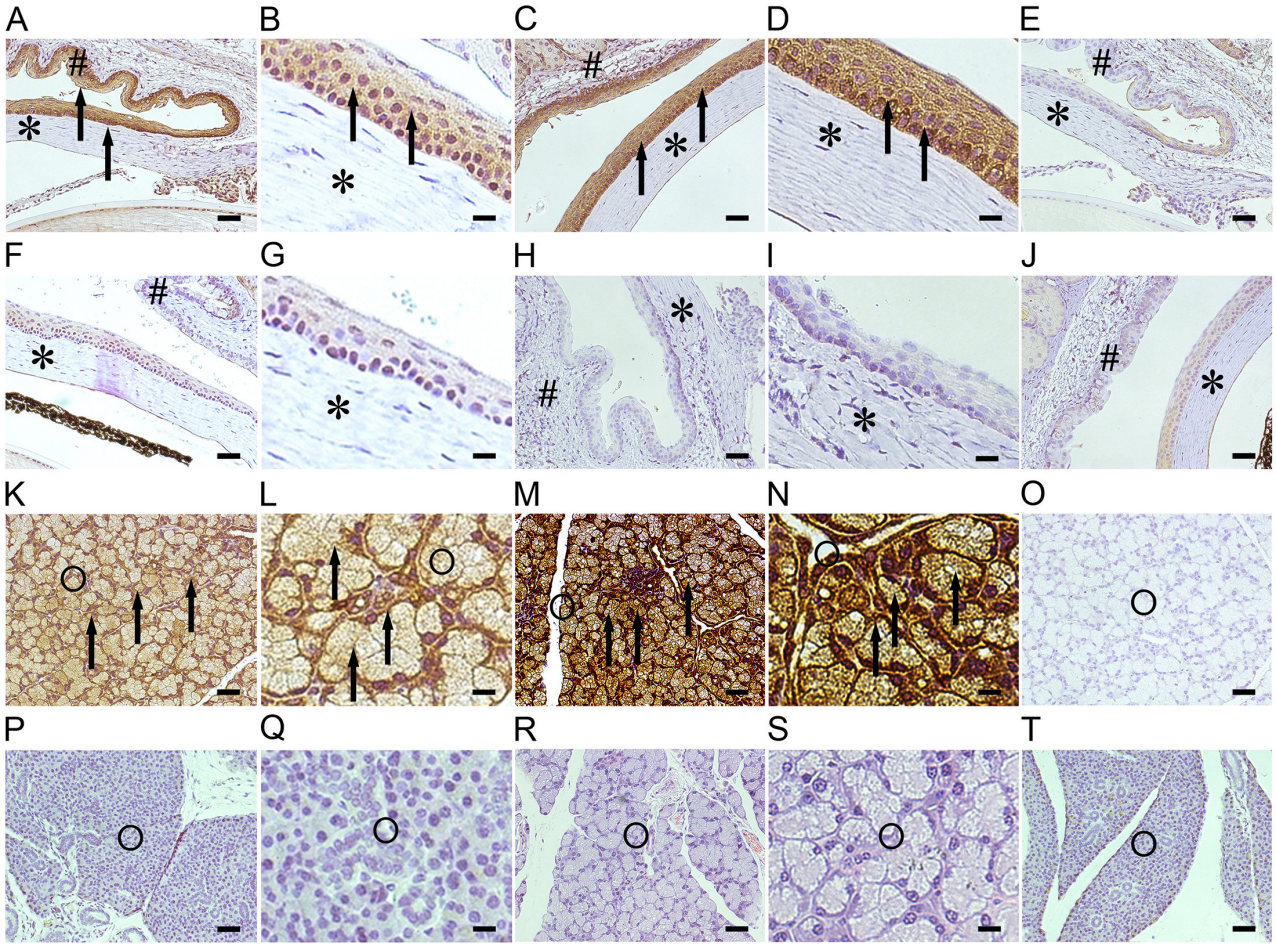
SP-D expression in the ocular tissues of WT and KO mice. SP-D expression in the ocular tissues was analyzed by immunohistochemistry (IHC) with SP-D antibody. SP-D expression was detected in the corneal epithelial and endothelial cells (A, B) and in acinar cells of the lacrimal gland in WT mice (K, L) (Pointed by arrows). In negative controls, which were processed using same protocol but without SP-D antibody incubation, they were negative in the cornea (E) and lacrimal gland (O). As expected, SP-D KO mice were negative for SP-D expression in the cornea (F, G) and lacrimal gland (P, Q) in the IHC with SP-D antibody, as well as negative controls of SP-D KO mice were negative in the cornea (J) and lacrimal gland (T). In addition, increased SP-D expression in the ocular epithelial and endothelial cells (C, D) andacinar cells of lacrimal gland (M, N) (pointed by arrows) were observed after bacterial challenge for 24 hours. SP-D KO mice were negative for SP-D expression in the cornea (H, I) and lacrimal gland (R, S) after inoculation with bacteria for 24 hours. Scale bars: (A, C, E, F, H, J, K, M, O, P, R, T) 50 μm, (B, D, G, I, L, N, Q, S) 20μm.The figures shown are from representative results in three independent experiments

SP-D gene knockout in KO mice was confirmed by PCR analysis of eyeball DNA (Fig 1). SP-D expression was studied in the ocular tissues of WT and SP-D KO (control) mice using IHC analysis as described in the Methods. The results demonstrate that ocular epithelial and endothelial cells were positive for SP-D expression in WT mice (Fig 2A and 2B). Strong positive staining signal of SP-D expression was detected in acinar cells of the lacrimal gland in WT mice (Fig 2K and 2L). As expected, negative controls (Fig 2E, 2J, 2O and 2T), as well as tissues from SP-D KO mice were negative for SP-D expression (Fig 2F, 2G, 2P and 2Q).In addition, increased level of SP-D expression in the ocular epithelial and endothelial cells (Fig 2C and 2D) and acinar cells of the lacrimal gland (Fig 2H and 2I) was observed after bacterial challenge.

### *S*. *aureus bacteria* were rapidly cleared from the ocular surface of WT mice compared to SP-D KO mice

**Fig 3:**
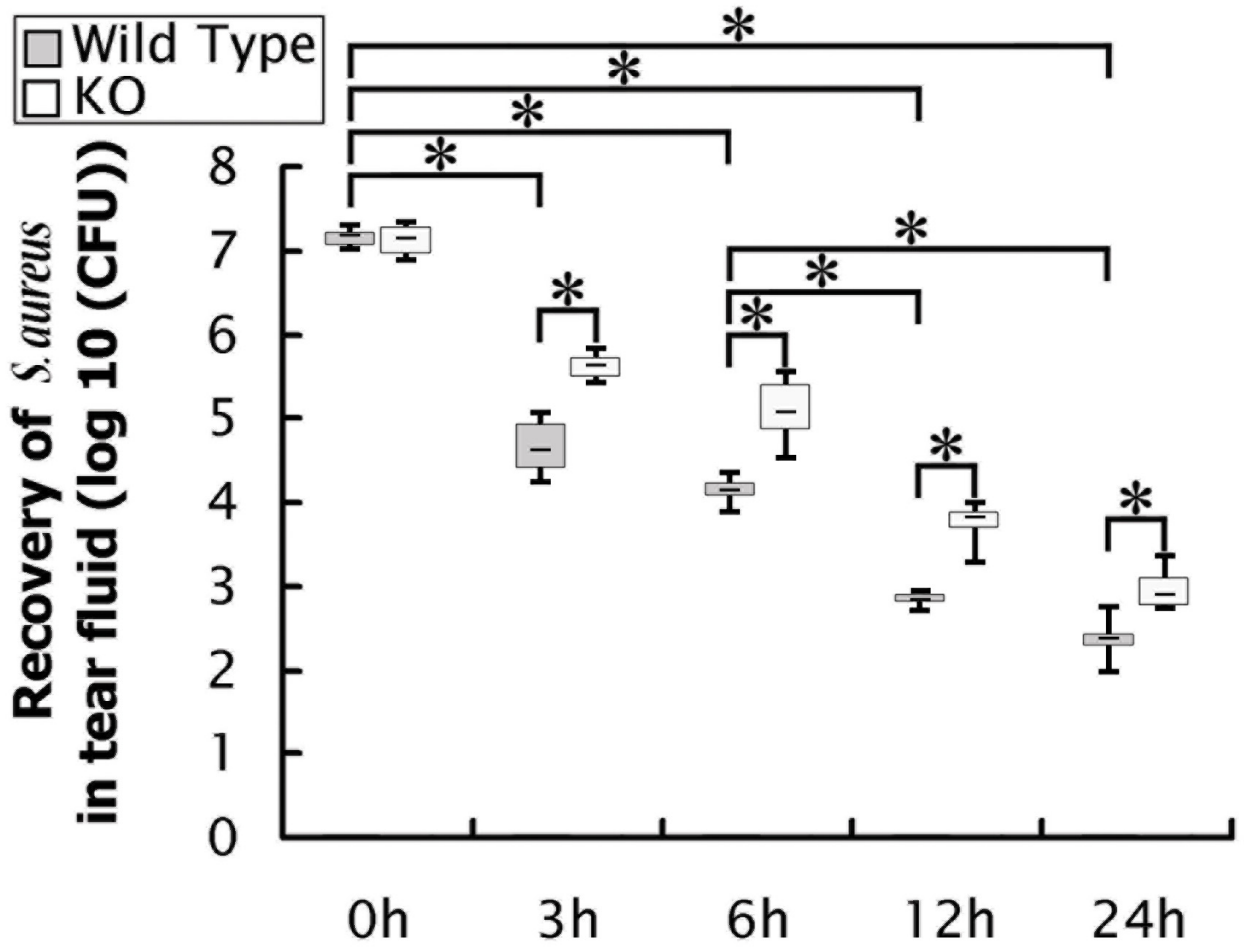
*S*. *aureus* bacteria were efficiently cleared from ocular surface of WT mice compared to SP-D KO mice. The CFUs recovered from the tear fluid of infected WT mice were examined at 3, 6, 12 or 24 h post-inoculation with *S*. *aureus* (10^7^ CFU/eye). Rapid clearance of the bacteria was in time-dependent manner. The CFUs of *S*. *aureus* from the ocular surface in infected WT and SP-D KO mice were compared at 3, 6, 12, and 24 h after inoculation of 10^7^ CFU/eye. A significant difference of the recovered bacteria in the tear fluid exists between infected WT and SP-D KO mice after infection. The results were from three independent experiments (n = 8 to 10 mice per group). Data are shown as the median (central black bar in boxes) with upper and lower quartiles (boxed area), and range of the data (error bars).* p<0.05 in Mann-Whitney U test.

The clearance of *S*. *aureus* was assessed by counting bacterial CFUs in the tear fluid collected from infected WT mice that ocular surface was inoculated with 10^7^ CFU of bacteria. A rapid decrease of CFUs in the tear fluid was observed in a time-dependent manner (Fig 3), suggesting that healthy eyes of WT mice could efficiently eliminate *S*. *aureus*. To study the role of SP-D in the clearance of *S*. *aureus*, SP-D KO mice were inoculated with 10^7^ CFUs/eye. The results showed that SP-D KO mice had significantly higher CFUs of bacteria (p<0.05) in tear fluid compared with WT mice at 3, 6, 12 and 24 h after infection (Fig 3). These indicated that SP-D is one important factor in the clearance of *S*. *aureus* from ocular surface.

### The clearance of *S*. *aureus* in mouse eyes was impaired by bacterial cysteine protease

**Fig 4:**
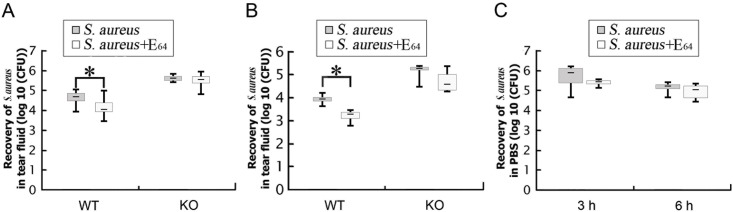
Cysteine protease inhibitor E_64_ increased the clearance of the bacteria on the ocular surface in infected WT mouse but not in SP-D KO mice. The CFUs of bacteria recovered from the tear fluid were examined at 3 h (A), and 6 h (B) after inoculation with *S*. *aureus* (10^7^ CFUs/eye) in infected WT and SP-D KO mice in the presence or absence of 10 nM of cysteine protease inhibitor (E_64_). The CFUs of bacteria were significantly decreased (p<0.05) in the tear fluid of the infected WT mice in the presence of E_64_ compared to its absence at both 3 and 6 h after infection. But no difference was observed in infected SP-D KO mice between the presence and absence of E_64_. Furthermore, to examine whether bacterial growth was influenced by the presence of E_64_, bacteria (10^6^ CFUs of *S*. *aureus*) were cultured in PBS with or without 10 nM of E_64_ for 3 and 6 h and then bacterial CFUs were assessed (C). The results showed no difference of CFUs with and without E_64_. The results shown are from three independent experiments (n = 8 to 12 mice per group). Data are shown as the median (central black bar in boxes) with upper and lower quartiles (boxed area), and range of the data (error bars).* p<0.05 in Mann-Whitney U test.

To examine the effects of extracellular proteases secreted from *S*. *aureus*, a selective-synthetic cysteine protease inhibitor (E_64_) was used in this study. The eyes of WT and SP-D KO mice were infected with 10^7^ CFUs/eye of *S*. *aureus* in the presence or absence of the inhibitor (10 nME_64_). After 3h (Fig 4A) and 6h(Fig 4B) inoculation, more bacteria were recovered in the tear fluid of the WT mice in the absence of E_64_ compared to the presence of E_64_. However, no significant difference was observed in SP-D KO mice between the presence and absence of E_64_. Furthermore, to examine whether the observed effects were caused by E_64_ directly killing to *S*. *aureus*, 10^6^ CFUs of the bacteria was incubated in PBS with 10 nM of E_64_ for 3 and 6 h and then total CFUs of *S*. *aureus* were analyzed. The results showed that there was no difference in CFUs of *S*. *aureus* between the presence and absence of E_64_ (Fig 4C). These results demonstrate that one potential mechanism of cysteine protease of *S*. *aureus* is to protect the bacteria from being cleared from ocular surface by neutralizing SP-D function.

### SP-D could enhance bacterial phagocytosis by neutrophils but cysteine protease of *S*. *aureus* impaired SP-D activity in the eye

**Fig 5:**
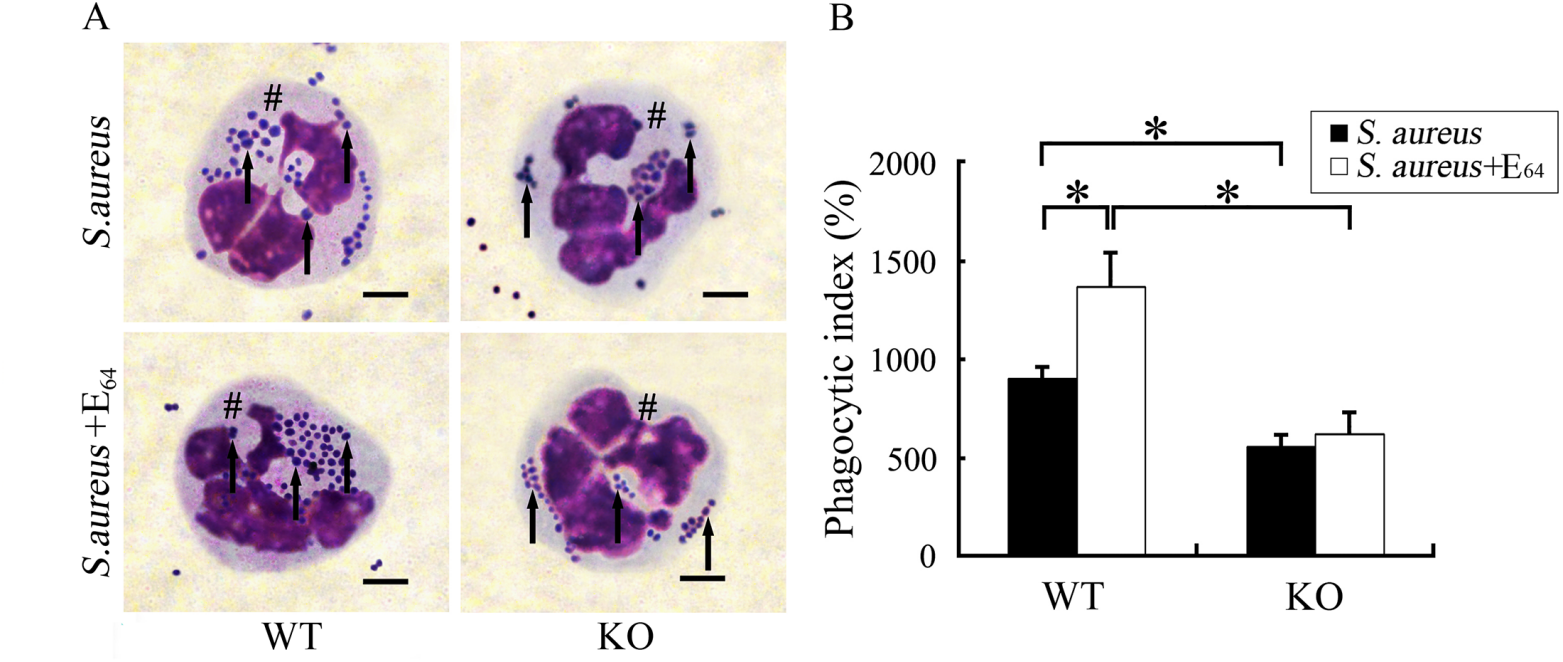
SP-D promoted *S*. *aureus* phagocytosis by neutrophils in ocular surface, but cysteine protease impaired SP-D activity. After inoculation with 10^7^ CFUs of bacteria or bacteria with 10nM E_64_ for 6h, tear fluid was collected and the cells were mounted on slides by cytospin centrifugation method. The cells were stained and one hundred randomly selected neutrophils per slide were analyzed at x1,000 magnification. Neutrophils that phagocytized at least one bacterium were counted as bacteria-positive neutrophils. The phagocytic index (PI) was calculated as the percentage of bacteria-positive neutrophils multiplied by the average number of bacteria per bacteria-positive neutrophils. Panel A shows neutrophils with bacteria stained using the Hema-3 Stain Kit. Panel B shows PI in infected WT and SP-D KO mice. “#” marked neutrophils. Arrows point to *S*. *aureus*. Scale bars: 3 μm. The results shown are from three independent experiments (n = 8 to 12 mice per group). All values represent mean ± SE. * p<0.05 in student’s t test.

To assess the effect of SP-D on bacterial phagocytosis by neutrophils, the phagocytic index (PI) was calculated (see methods). Cells were prepared from tear fluid collected from infected mice and fixed on slides by cytospin centrifugation. Bacterial phagocytosis by neutrophils was evaluated in both infected WT and SP-D KO mice using microscopy (Fig 5A). After inoculation with *S*. *aureus* for 6h, the PI was higher in WT mice than SP-D KO mice (p<0.05) (Fig 5B). In the presence of cysteine protease inhibitor E_64_ (10nM), the PI was significantly increased (p<0.05) in WT mice but not in SP-D KO mice (Fig 5B). These demonstrate that SP-D could promote *S*. *aureus* phagocytosis by neutrophils in ocular surface but cysteine protease reduced SP-D activity.

### SP-D could reduce *S*. *aureus* invasion in epithelial cells but bacterial cysteine protease impaired SP-D activity

**Fig 6:**
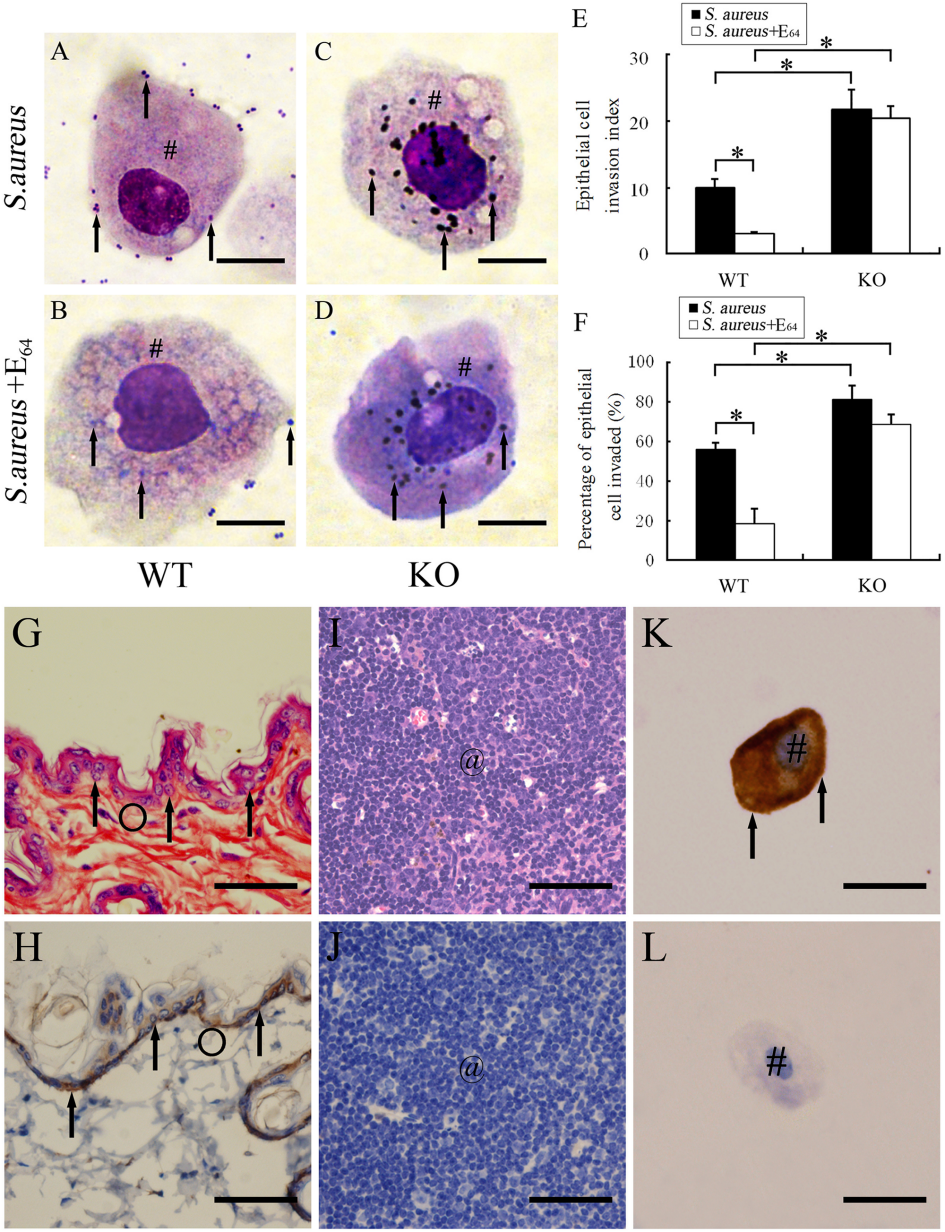
SP-D inhibited *S*. *aureus* invasion in ocular epithelial cells but *S*. *aureus* cysteine protease impaired SP-D activity. After a 6-h inoculation of *S*. *aureus*, tear fluid sedimentation was applied to slides by cytospin centrifugation. **Panel one (A-F)**: The slides were stained and analyzed by light microscope under oil immersion(X1000). Adherence and internalization steps of bacteria for each condition (A-D) were counted as invaded bacteria (arrow). The invasion index (E) and percentages of invaded epithelial cells (F) were calculated, as described in the Methods. Invasion index of invaded bacteria in KO mice was higher (p<0.05) compared with WT mice. E_64_ treatment could reduce the index in WT mice but not in SP-D KO mice. **Panel two (G-L)**: To confirm epithelial cells collected from mouse tear fluid, immunohistochemistry and immunocytochemistry analyses were performed with pancytokeratins AE1/AE3. (G) Hematoxylin and eosin (H&E) staining of murine skin. Arrow shows epithelial cell. (H) Positive control. Immunohistochemistry revealed the epithelial cells (arrow) of murine skin to be positive for pancytokeratin AE1/AE3. (I) H&E staining of murine lymph nodes. (J) Negative control. Immunohistochemistry indicated the lymph node to be negative for pancytokeratin AE1/AE3. (K) Immunocytochemistry revealed a positive immunoreaction for cytokeratin AE1/AE3 was present throughout the cytoplasm of the epithelial cells in tear fluid (arrow). (L) Negative control. Same protocol was applied but the primary antibody was replaced with isotype-matched goat IgG. Scale bars: (A-D) 10 μm, (G-J) 100μm, (K-L) 20 μm. WT, WT mice. KO, SP-D KO mice. Cycles indicate murine skin. “#” and “@” mark murine epithelial cell and lymph node, respectively. The results are from three independent experiments (n = 8 to 12 mice per group).All values represent mean ± SE. * p<0.05 in student’s t test.

To determine whether SP-D contributed to the mechanism of ocular surface epithelial cell protection against *S*. *aureus* invasion, invasion index of epithelial cells was studied. A representative photo for each condition was shown in Fig 6A and 6D. Epithelial cells invasion index was significantly increased in KO mice (p<0.05) at 6 h after inoculation with 10^7^ CFUs of *S*. *aureus* compared with WT mice (Fig 6E). Similar results were observed in percentage of epithelial cells invaded by bacteria in WT and SP-D KO mice (Fig 6F). We also examined the effect of cysteine protease inhibitor E_64_ on bacterial invasion in epithelial cells *in vivo*. The invasion index and percentage of epithelial cells invaded by bacteria in the presence or absence of 10 nM E_64_ was calculated. The results showed that the E_64_ treatment significantly decreased the adherence and invasion of *S*. *aureus* into epithelial cells of ocular surface in WT mice (p<0.05), but not for SP-D KO mice (Fig 6E and 6F).

Furthermore, we performed IHC analysis to confirm epithelial cells collected in tear fluid with pancytokeratins AE1/AE3 (a biomarker of epithelial cells) antibody. As shown in the Fig 6K, epithelial cells showed positive immunoreactions for pancytokertins AE1/AE3. As expected, the negative control and spleen tissues were negative (Fig 6J and 6L).

### Ocular surface injury was more severe in infected SP-D KO mice compared to infected WT mice

**Fig 7:**
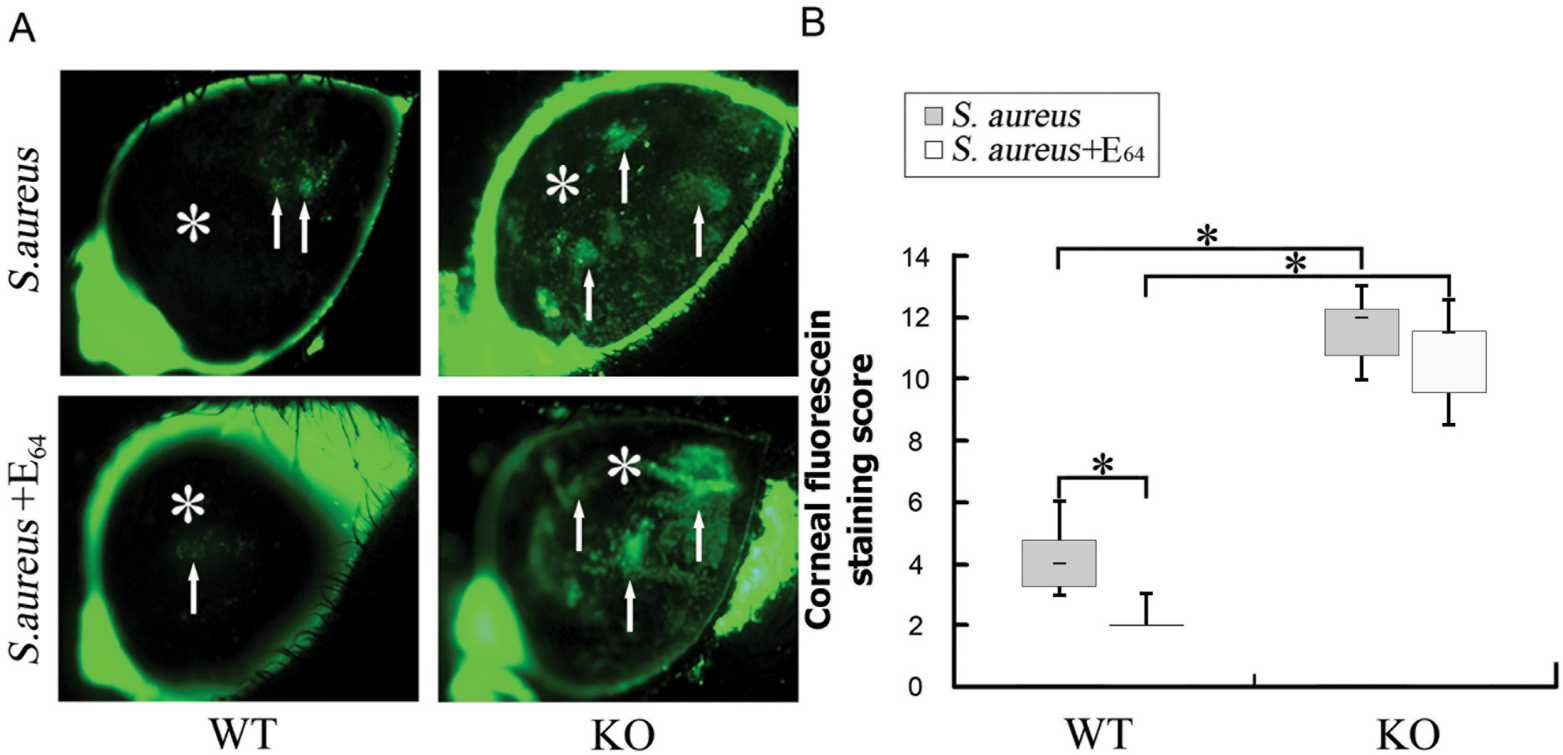
Comparison of eye injury of WT and SP-D KO mice after *S*. *aureus* infection in the presence or absence of E_64_. Mouse corneal integrity (eye injury) was examined using fluorescein staining method after inoculation of *S*. *aureus* for 24 h. The results show increased fluorescein staining (arrows) in the cornea of SP-D KO mice compared to WT mice. E_64_ treatment could reduce fluorescein staining in WT mice but not in SP-D KO mice. Corneal fluorescein staining scores in SP-D KO mice were higher than that in WT mice (B). WT, WT mice. KO, SP-D KO mice. The figures shown are from three independent experiments (n = 8 to 10 mice per group). Data are shown as the median (central black bar in boxes) with upper and lower quartiles (boxed area), and range of the data (error bars).* p<0.05 in Mann-Whitney U test.

Corneal integrity/ocular surface injury was assessed by fluorescein staining at 24 h after challenging with *S*. *aureus*. Based on the signal of fluorescein staining ocular surface injury in infected SP-D KO mice was more severe compared to infected WT mice (Fig 7A). Similar results were observed in corneal fluorescein staining scores which were higher in SP-D KO mice than that in WT mice (Fig 7B). In the presence of inhibitor E_64_ ocular injury (fluorescein staining) was reduced in infected WT mice, but not in infected SP-D KO mice (Fig 7A and 7B).

### SP-D levels in tear fluid decreased by *S*. *aureus* cysteine protease

**Fig 8:**
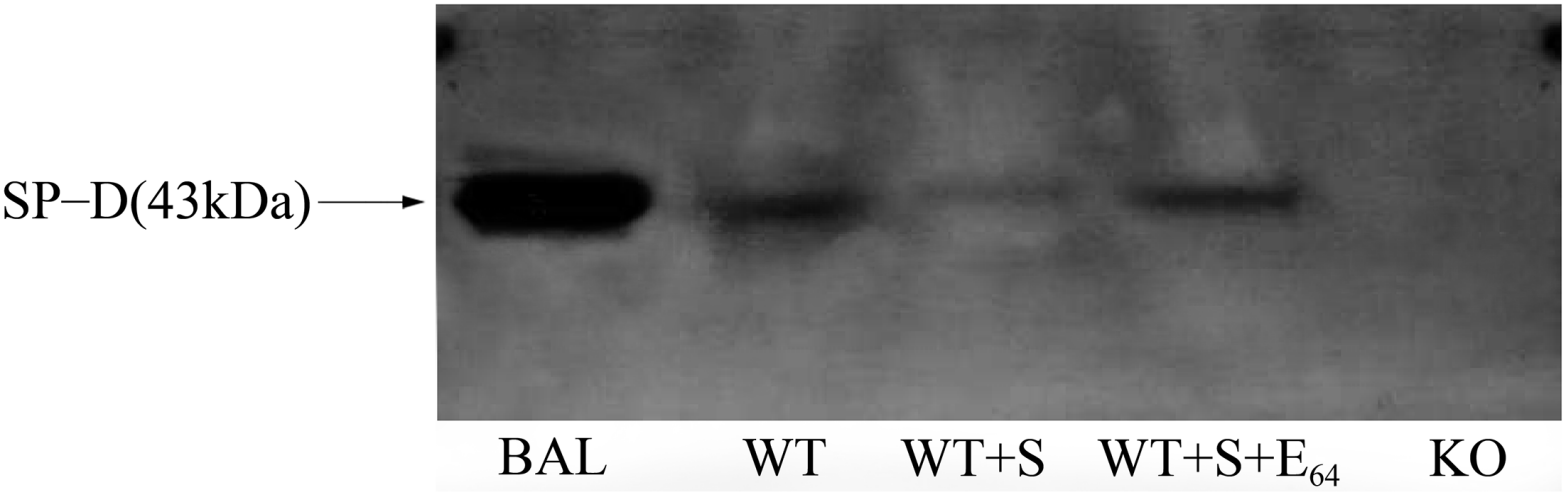
Western blotting analysis of SP-D level in mouse tear fluid. SP-D levels in the mouse tear fluid were examined by Western blot analysis. Mouse tear fluid was collected and pooled from five WT mice. The tear fluid was incubated with *S*. *aureus* conditioned medium in the presence or absence of 10 nM E_64_ for 1 h. After Western blot analysis with SP-D antibody, a band of SP-D (about 43 KDa) was detected in mouse tear fluid (Lane 2), but the level of SP-D in the tear fluid with *S*. *aureus* conditioned medium was reduced (Lane 3) compared to the tear fluid without *S*. *aureus* conditioned medium (lane 2). In the presence of inhibitor E_64_, SP-D level of the sample (Lane 4) has similar to the tear fluid sample (Lane 2), suggesting that decreasing of SP-D level was inhibited by E_64_. Mouse lung BAL fluid was used as SP-D positive control (Lane 1). As expected, the tear fluid from SP-D KO mice did not show an SP-D band (Lane 5). WT,WT mice. KO, SP-D KO mice.BAL, lung bronchoalveolar lavage. Three independent experiments were performed.

To examine whether extracellular cysteine protease of *S*. *aureus* causes a reduction in SP-D levels, the tear fluid from WT mice was incubated with *S*. *aureus* conditioned medium in the presence or absence of E_64_. SP-D levels were examined by Western blotting analysis. As shown in Fig 8, SP-D protein was remarkably reduced when mouse tear fluid was incubated with *S*. *aureus* conditioned medium, but this reduction was attenuated in the presence of cysteine protease inhibitor E_64_. These data suggest that cysteine protease of *S*. *aureus* was a factor which might cause the reducing of SP-D levels in tear fluid.

## Discussion

In the present study we demonstrate that SP-D contributes to the rapid clearance of *S*. *aureus* from the ocular surface and plays a protective role in *S*. *aureus* eye infection. We identified SP-D expression in ocular surface epithelial and endothelial cells and in the lacrimal gland of mouse eyes. SP-D could enhance bacterial phagocytosis by neutrophils and decrease bacterial invasion into ocular epithelial cells; SP-D could also maintain corneal epithelial integrity and reduced ocular surface injury. Furthermore, we found that cysteine protease secreted from *S*. *aureus* is one major factor that decreased SP-D levels and function against bacterial infection in the eye.

In this study WT mice exhibited rapid clearance of *S*. *aureus* in healthy ocular surface in comparison to SP-D KO mice, suggesting that SP-D plays an important role against *S*. *aureus* in eye infection. Previous works demonstrated that several antimicrobial compounds in tears such as lysozyme, lactoferrin, lipocalin, secretory immunoglobulin A (IgA) and complement play a role in killing Gram-positive bacteria [47]. SP-D, as one member of C-type lectin protein family, contains a carbohydrate recognition domain (CRD)that can bind to carbohydrate moieties on the surface of *S*. *aureus* in a Ca^2+^-dependent manner [13]. SP-D can also interact with lipoteichoic acid (LTA) and peptidoglycan (PepG), two major cell wall components of Gram-positive bacteria [48].The interactions between SP-D protein and bacterial components cause bacterial aggregation and enhance phagocytosis by macrophages and neutrophils. Furthermore, SP-D can promote activation of macrophages and neutrophils by enhancing the production of nitrogen species and superoxidative burst [13].

*S*. *aureus* keratitis was characterized by intensive neutrophil infiltration and bacterial invasion of the underlying stroma, which can result in corneal opacification and potentially loss of vision [49–54]. In the present work we observed a higher bacterial phagocytic index by neutrophils and reduced bacterial invasion index of epithelial cells in the WT mice compared to SP-D KO mice, indicating SP-D has a protective role in keratitis. Recent studies have shown that SP-D not only binds to a variety of bacteria, like *Pseudomonas aeruginosa* and *Escherichia coli*[55–57], but also directly inhibits bacteria invasion in non-phagocytic cells [11]. The results that SP-D inhibited *S*. *aureus* invasion into ocular surface epithelia in this study is consistent with other study [11]. Although ocular surfaces are constantly exposed to a various environmental factors including a variety of microbes, few of them are able to cause invasive infection. This is partially due to the formation of tight barriers in epithelial surfaces that are highly effective in blocking penetration by most microbes. Therefore, penetration of *S*. *aureus* through the barriers of the epithelial surfaces requires several key steps including attachment to the epithelial surface, transepithelial migration, and evasion of immune mechanisms. A previous study indicated that LTA, one of the major cell wall components of Gram-positive organisms, mediates the adherence of *S*. *aureus* to fibronectin on the epithelial cell surfaces [58]. The LTA induced disruption of the epithelial barrier and promotion of *S*. *aureus* invasion are in a Toll-like receptor 2 (TLR2)-dependent manner, in which TLR2 ligand activates p38 MAP kinase and transforms growth factor (TGF)-β signaling pathways. SP-D can bind to LTA of Gram-positive bacteria via its CRD [48], providing one possible mechanism to block bacterial LTA-mediated disruption of epithelial barrier in the eye. However, the detailed mechanisms still need to be studied in the future.

In the present study, the IHC analysis revealed that the SP-D was expressed in corneal epithelial and endothelial cells, as well as in lacrimal glands. The level of SP-D expression elevated after *S*. *aureus* infection. A previous study reported that exposure to LTA increased Surfactant protein A (SP-A) expression in human alveolar type II epithelial cells through sequentially activating the MEK1-ERK1/2-NF-κB-dependent pathway [59]. *S*. *aureus* LTA can induce host inflammatory response of the ocular surface, therefore increased SP-D expression in the epithelium of ocular surface after inoculation with *S*. *aureus* can elevate host innate immune ability, benefitting local host defense against bacterial infection in the eye. In contrast, SP-D KO mice lack SP-D’s protective role and thus SP-D KO mice exhibited more severe infection in the present study. In addition, we tried to determine the presence of another innate immune molecule SP-A. We failed to detect SP-A in the tear fluid in mice (data not shown) using Western blotting analyses, while SP-A could be detected in the human eye. This may be due to technical reasons or a lower level of SP-A expression in mouse tear fluid.

In this study, we observed that cysteine protease inhibitor could efficiently promote the *S*. *aureus* clearance from ocular surface in WT mice, but not for SP-D KO mice. The successful innate immune protection required a network of cellular and humoral factors like epithelial cells, phagocytes, cytokines, complement proteins, coagulation factors, and soluble pattern recognition molecules [60]. Initiation of these cell and molecular activation factors requires the involvement of pattern recognition molecules such as surfactant protein SP-A and SP-D. However, bacterial protease could degrade host immune molecules and abolish the function of these innate immune proteins, which would make bacterial protease a vital tool for bacterial resistance to host innate immunity. It was previously reported that *P*. *aeruginosa* protease, i.e. elastase, could degrade tear fluid SP-D *in vivo* and compromise the clearance of *P*. *aeruginosa* from the healthy ocular surface [8]. Recent study indicated that cleavage of SP-A by the cysteine protease of *S*. *aureus* resulted in the abolition of SP-A biological activity in bronchoalveolar lavage fluid [61]. In our study, we found that incubation with *S*. *aureus* conditioned medium decreased SP-D in tear fluid from WT mice, but cysteine protease inhibitor E_64_ could prevent SP-D degradation. The degradation of SP-D by cysteine protease in the ocular surface hampered bacterial clearance, while E_64_ inhibited the function of cysteine protease in cleavage of SP-D and increased the clearance of *S*. *aureus*. Furthermore, we also found that E_64_ interfered with bacterial adherence to and invasion of epithelial cells and reduced the damage of corneal barrier function, suggesting that cysteine protease favors symbiosis of *S*. *aureus* with the host that makes it an important colonization factor. However, the detailed mechanisms are not well understood. For example, how does cysteine protease degrade SP-D in tear fluid *in vivo*? How does cysteine protease influence bacterial adherence to, invasion in and penetration through corneal epithelial cells, and elevate bacterial colonization in cornea?

In summary, this murine model demonstrates that SP-D, as an important innate immune molecule, could enhance the ocular clearance of *S*. *aureus* and reduced ocular surface injury from bacterial infection. Furthermore, cysteine proteases from *S*. *aureus* caused decreasing levels of SP-D and impaired SP-D function. However, further investigation will be necessary to approach the detailed mechanisms of SP-D innate immunity in ocular surface infection, which may lead to the development of new therapies for bacterial eye infections such as *S*. *aureus* keratitis.
